# Reply to a Case of Instrumental Delivery

**Published:** 1868-04-15

**Authors:** E. K. Travers

**Affiliations:** Amboy, Ill.


### REPLY TO A CASE OF INSTRUMENTAL DELIVERY

*Reported by W. Anderson, M.D., Leroy, Ill.*

BY E. K. TRAVERS, M.D., AMBOY, ILL.

In the March number of the Journal, there is a case reported by W. Anderson, M.D., of Leroy, Ill., where he was called in consultation with a Dr. D., to a lady in labor, with a shoulder presentation, the head in the left acetabulum, the left arm protruding from the vagina, and very much swollen, so that the insertion of the hand into the uterus was impossible, while the force of the uterus was so great as to prevent an adjustment of the fœtus by bringing the head into the pelvis. The arm, he says, could not be replaced; and he decided, with Dr. D., an amputation unavoidable, which he performed at the shoulder. He also says that he knew the infant was dead before the operation; which, however, did not influence his action.

Now, not having any claim to be a sage practitioner, I suppose I ought to remain dumb; but having had the experience of several such cases, during the last few years, I dare to reply. In the first place, my practice is to put my patient completely under the influence of *Chloroform*, thus quieting the force of the uterus, which allows me to slip my hand up by degrees, persistently resisting all forces, and never withdrawing my hand until after I take hold of the child's feet, and draw them gradually into the world; while the other hand is on the abdominal parieties of my patient, assisting to turn the child. Now, I think there is no necessity whatever to mutilate the child, in such a presentation. I don't consider the arm at all in the way, in turning the child and delivering by the feet; but I do think it useless to try to bring the head into the pelvis, or replace the arm, as I think it can not be done in such cases. My motto is, not to waste precious time in useless endeavors.

The following is the last case of the kind I had: Aug. 25th,

1867, I was called, about 6 P.M., to attend Mrs. T. F., in her third confinement. Labor had already progressed for twelve hours. The membranes had ruptured at 5 A.M., previous to my arrival; and the amniotic fluid escaped. The head of the child was in the right iliac fossa, with the left arm and shoulder presenting, the back of the child directed towards the abdomen of the mother. The arm was very much swollen, and presenting in the vulva. To complicate the case more than this one Dr. Anderson quotes, this woman had a very narrow and contracted pelvis; her first child, which was still-born, had to be taken from her with forceps, after a long and protracted labor of eighteen hours. The cranium was very much compressed, and elongated in coming through the pelvis; but I desisted from using the forceps. Taking these facts into consideration, I made up my mind that I had a very difficult case; and I sent for my friend, Dr. Felker, to whom I am very much indebted for his efficient aid in turning and delivering the child, after first putting her completely under the influence of *Chloroform*. Before the anæsthetic was given, it was impossible to introduce the hand, from the contracted state of the womb. I quote this case, from its similarity to that of Dr. Anderson's.

In connection with this subject, I would add that there is too much and too early an interference with natural labors, with too great a desire to turn them into instrumental ones; and when I think how many innocents are ruthlessly destroyed by either inconsiderate or incomplete accouchers, I shudder. A couple of weeks ago, in this locality, there was a lady in labor for about twelve hours, with a very natural presentation. The labor had progressed until the occiput was presenting at the vulva, with the head pressing on the perineum; when the young man in attendance, without consultation, performed the operation of craniotomy — and then boasted of a great operation! But if this was the only case of the kind, it would be well; but I know of several just such cases. I am sometimes inclined to think that men are induced to commit such uncalled-for operations, for the purpose of gaining a reputation for talents that they do not possess.

